# Supplementary material for: Are carbon emissions trading and green financial instruments synergistic? -Comprehensive quantitative research based on content analysis
Source: PLoS One. 2024 Mar 7;19(3):e0298601. doi: 10.1371/journal.pone.0298601 (PMC10919601; doi:10.1371/journal.pone.0298601)
Supplement: S1 Data — (ZIP) [file pone.0298601.s001.zip › Data/地级市Z_F工作报告.zip!/║┌┴·╜¡/║┌║╙╩╨/└┤╫╘╣1⁄2╓┌║┼í╛┬φ┐╦╩2╛▌═°í┐_2012.docx]

各位代表：

现在，我代表市人民政府向大会作工作报告，请予审议，并请各位政协委员和列席会议的同志提出宝贵意见。

一、2011年工作回顾和过去五年发展成就

2011年，在省委、省政府和市委的正确领导下，我们紧紧依靠全市人民，深入贯彻落实科学发展观，创新工作思路，狠抓工作落实，全面完成了市四届人大六次会议确定的任务目标。预计全市生产总值300亿元，增长13%。全社会固定资产投资155亿元，增长30%。社会消费品零售总额64亿元，增长17%。全口径财政收入30亿元，增长22.4%。城镇居民人均可支配收入14800元，农村居民人均纯收入8650元，分别增长19%和22.8%。万元地区生产总值综合能耗下降3%。一年来，主要做了以下工作：

——全力以赴抓开工、抢进度，项目建设在攻坚克难中实现突破。开展“产业项目建设年”活动，重点项目开复工79项，完成年度投资185亿元，增加60亿元，增长48%，其中，产业项目开复工48项，完成投资58.3亿元，增长85.7%，创历史最好水平，成为推动发展的强劲支撑。嫩江多宝山铜矿、嵩天薯业淀粉加工、北安上海电气风电设备制造等项目竣工，爱辉区龙源风电并网发电，五大连池风景区40万吨矿泉水试生产，合作区龙江化工聚乙烯醇、合盛多晶硅项目具备投产条件，孙吴大果沙棘、逊克东安岩金矿、北安汽车产业园等项目加快推进，爱辉区燕麦深加工、五大连池北大荒矿泉水等项目开工建设。园区平台作用进一步发挥，集聚功能不断提升，一大批产业项目建成投产，推动了工业经济优化升级，规模以上工业增加值增长16%。千方百计克服征地和资金困难，公路建设“三年决战”全线告捷，吉黑高速北安至黑河段、绥北高速北安段、加黑公路嫩江星火九连至黑河段等工程建成通车，嫩黑公路基础完工。新建公路1300公里，农村公路488公里，总里程分别达到1.4万公里和3945公里，覆盖城乡的交通网络基本形成。

——坚定不移调结构、强基础，现代农业在局市共建中亮点纷呈。农业增加值增长12%。粮食总产335万吨，增长27%，再创历史新高。农业机械化程度位居全省前列，共组建农机合作社227个，机械化率达到95%。加快土地承包经营权流转，规模经营面积过半。农民组织化程度显著提高，新建专业合作社55个，带动10万农户，辐射876万亩耕地。水利建设迎来新发展，经积极争取，各县（市、区）全部纳入国家小型农田水利重点县，为今后发展赢得了持久资金支持。结构调整取得重大进展，大豆面积减少92万亩，寒地水稻试种成功，玉米实现高产。林业特色产业加快发展，中俄林业科技合作园区成功引进蓝靛果忍冬和花楸等品种，繁育面积达1800亩。对俄农业合作不断深化，境外土地开发近百万亩。畜牧标准化养殖水平明显提升，3家养殖场列入省级示范创建场。网箱养殖带动渔业快速发展。局市共建经验在全省推广，完成“三代”面积712万亩，建设农业科技园区25个，垦地合作更加紧密。

——拓宽视野谋共识、促交流，对俄合作在互利共赢中不断深化。对外贸易进出口总值32.8亿美元，增长15%。口岸出入境112万人次，增长8%。“两国一城”发展理念首次在黑河与阿州政府层面达成共识，确定了能源、旅游、通道建设等8件大事，奠定了深化合作的坚实基础。对俄合作项目取得重大进展，浮箱固冰通道当年开工、当年建成，延长了冬季通关时间。阿穆尔—黑河边境油品储运与炼化综合体项目国内所有支持性文件全部完成。对俄文化交流实现新突破，第二届中俄文化大集成功举办，得到省政府充分认可，纳入我省与阿州文化交流机制项目落户黑河。基层政党交流更加紧密，民间组织交流日趋活跃，对俄桥头堡地位进一步巩固。招商引资成效显著，落实招商项目122项，到位资金81.7亿元，增长12.2%。

——坚持不懈打品牌、聚人气，消费经济在旅游牵动中急剧升温。接待国内外旅游者315万人次，实现收入42亿元，其中，五大连池风景区旅游人次突破120万。加大宣传营销力度，在满洲里首届中俄地区旅游合作交流会、杭州世界休闲博览会上，“中俄双子城”品牌引起广泛关注。强化央视主流媒体宣传，城市知名度明显提升。五大连池“圣水节”列入国家非物质文化遗产名录。五大连池风景区名镇建设加快推进，火山博物馆完成前期，机场预可研通过评审。五大连池温都水城国际酒店投入运营，黑河龙江国际交流中心开工建设。试车经济创出品牌，被国家汽协确定为寒地试车基地。会议规模和层次实现突破，承办了全国人大新闻工作会议、首届中国电视发展年会等国家级会议，聚集人员增长20%。

——创新理念抓建设、强管理，城市品位在提档升级中彰显特色。立足“铸魂、提品、增绿、静音”，开展 “三城联创” 活动，“三优”文明城市建设不断深化。全市城建基础设施投资6.2亿元，其中，市区2.7亿元，新建改造道路21条，道路完好率98%。市容市貌和人居环境明显改善，新增绿地8.5万平方米。黑龙江公园四期工程启动。改造临街建筑立面103栋，亮化楼体41栋。改造住宅小区63个，维修老旧住宅楼203栋，受益居民达1.1万户。全市改造棚户区125.7万平方米，超额完成省计划。北安、嫩江、五大连池和风景区东区污水处理厂建成，黑河、嫩江、孙吴生活垃圾处理厂完工。城市管理进一步加强，数字化城市建设加快推进，王肃街、龙江路等主要路段试行重点车型“限行”。

——千方百计壮财源、保重点，财税金融在推动发展中提升水平。全市一般预算收入18.4亿元，一般预算支出94.6亿元，分别增长22.6%和10.5%。市直一般预算收入5.2亿元，一般预算支出15.4亿元，分别增长38.7%和10.9%。加快财源项目建设，合作区被批准为省级财源建设示范区。支出结构进一步优化，就业、教育、社保支出不断提高。完善监管机制，投资评审、政府采购和资金管理工作明显加强。农村商业银行成功组建。一期8亿元城投债券进入发行程序。与省工商行等金融机构签订150亿元融资合作协议。人民银行黑河支行启动了“两管理、两综合”及金融消费者权益保护工作。

——真心实意惠民生、解民忧，群众福祉在力促和谐中持续改善。实施34项惠民行动，民生“十件实事”全部完成。城镇新增就业2万人，登记失业率3.74%。大力实施30周岁以下高校毕业生就业工程，安置3274人。启动城镇居民养老保险试点和农村新型养老保险第二、三批试点，提高城乡社会保险待遇水平和最低生活保障补贴标准。海澜热电厂新增供热能力200万平方米，可满足市区今后三年供热需求。实施省级以上科技项目14项，建设公办幼儿园20所。市中医院新建工程投入使用，市第一人民医院晋升为三级乙等医院。黑河儿童福利院和流浪未成年人救助中心建成使用。高度重视安全生产，连续11个防期未发生重特大森林火灾。完善社会矛盾调处机制，加强社会治安综合治理，社会秩序保持稳定。边境管理进一步加强。军政军民关系和谐发展，“双拥模范城” 创建成果进一步巩固。计生、老区、民族宗教、地震、侨务、妇女儿童、残疾人、人防、档案、地方史志等工作取得新成绩。

——健全机制提效率、树形象，执行能力在创新实践中明显增强。主动接受人大依法监督和政协民主监督，138件建议和提案全部办结。大力倡导“五讲五重”，制定政府工作规则，完善重大事项决策公示、听证和专家咨询论证制度，建立重大决策实施情况跟踪反馈制度。完善突发公共事件应急体系。加快电子政务建设。开展加强机关作风建设和提高效能专项活动。严格执行廉洁从政规定，党风廉政建设进一步加强。

各位代表，过去一年取得的丰硕成果，为本届政府工作画上了圆满句号。回顾刚刚过去的五年，我们在应对金融危机中寻找契机，在抢抓政策机遇中赢得先机，在不断破解发展难题中阔步前行，经济社会取得显著成就，谱写了改革发展的新篇章。五年来，致力产业升级，力抓方式转变，发展质量不断提升。始终坚持产业引领发展，努力实现加快发展与优化结构、提升效益的有机统一。种植结构调整取得历史性突破，粮食产量五年翻一番。牢牢抓住投资增长不动摇，紧紧扭住产业发育不懈怠，建成和在建重点项目153项。园区承载能力和集聚功能不断增强，工业经济对财政贡献率大幅提升。以旅游为龙头的消费经济持续发力，现代服务业成为新的经济增长点。非公经济迅速壮大，增加值突破80亿元，在推动发展、促进就业方面发挥了重要作用。加大统筹力度，力求区域协调，城乡面貌大为改观。始终坚持市县同步提档、城乡一体建设、地方农垦联动，加快推进区域发展。我市整体纳入国家主体功能区、大小兴安岭林区转型规划，边境县（区）均列入兴边富民支持范围，爱辉区、逊克、五大连池争得资源枯竭城市转移支付政策，为今后发展积攒了后劲。城镇化步伐加快，城乡功能不断完善，“三优”文明城市创建走在全省前列。注重机制创新，力推改革开放，发展活力持续增强。始终坚持把改革开放作为推动发展的强大动力，积极推进重点领域和关键环节改革，激发出更大的发展活力。在土地流转、林权制度、招商引资、城市管理、森林防火、机构改革等方面，不断探索符合黑河实际的新模式。大力发展外向型经济，对俄贸易进出口总值累计实现50亿美元，实际利用外资2亿美元。践行以人为本，力解民生难题，社会事业全面发展。始终坚持发展为了人民、发展依靠人民、发展成果由人民共享。教育、医疗基础设施持续改善，文化、体育事业繁荣发展。社会保障体系更加健全，就业渠道不断拓宽，人民生活水平大幅提高。城镇居民人均可支配收入、农村居民人均纯收入分别增长1.6倍和1.5倍。加强依法行政，力优发展环境，行政效能不断提升。始终坚持办事讲程序、工作讲规则、决策讲民主，加快职能转变，改进工作作风，增强执行力和公信力。开展行政执法权力清理，行政复议案件办理质量明显提高。建立健全政府网站、行风评议、市长热线、信访大厅等互动平台，自觉接受公众监督。

各位代表，过去的五年，是黑河历史上经济增速最快、社会事业发展最好、城乡面貌变化最大、人民群众得到实惠最多的时期。全市生产总值翻一番，全社会固定资产投资、全口径财政收入翻两番以上。这些成就的取得，是全市上下坚定信念、精诚团结、顽强拼搏、干事创业的结果。市委统揽全局、协调各方，牢牢把握科学发展的方向。市人大创新工作方式，通过绩效考核助推政府工作提速。市政协积极参政议政，集中委员智慧服务政府科学决策。中省直各部门以地方发展为己任，全力支持和落实政府重大部署。方方面面给予政府的鼎力支持和无私帮助，我们铭记在心。在此，我代表市人民政府，向辛勤工作在各行各业的全市人民，向各级人大代表、政协委员和各民主党派、工商联、无党派人士、人民团体，向关心支持地方工作的中省直单位和人民解放军、武警官兵，表示衷心的感谢，并致以崇高的敬意！

取得的成就令人鼓舞，发展的历程充满艰辛，把握过去才能更好地开创未来。我们在看到成绩的同时，也清醒地认识到存在的问题。主要是：经济总量小，财政实力弱，发展内生动力不足。经济结构不合理，产业项目规模小，农业产业化进程缓慢，工业体系还不健全。进出口加工企业少，外贸对财政贡献率低。个别部门和干部用市场经济方法解决实际问题能力不强，精神状态、工作作风与发展要求还不相适应等等。对于这些问题，我们一定要以高度负责的态度，采取有效措施，认真加以解决。

二、未来五年发展愿景

未来五年，是黑河加快发展、加速崛起的重要时期。面对复杂多变的国际经济环境和国内经济运行的新情况新变化，迫切要求我们树立战略思维，不断提高应对复杂局面、驾驭发展的能力。面对主体功能区、兴边富民、沿边开放等国家战略的进一步实施，迫切要求我们增强机遇意识，不断提高抢抓发展先机、引领发展的能力。面对历届班子打下的良好基础和未来发展的繁重任务，迫切要求我们弘扬创新精神，不断提高敢于打破常规、跨越发展的能力。面对区域发展不平衡、不协调，社会建设相对滞后和社会管理不适应问题，迫切要求我们强化民本思想，不断提高调处社会矛盾、和谐发展的能力。站在新起点，面对新任务，我们应进一步找准黑河发展的历史方位，切实增强工作前瞻性，牢牢把握发展主动权，努力追求卓越，实现跨越赶超。

未来五年，我们要按照市五次党代会的要求，高举邓小平理论和“三个代表”重要思想伟大旗帜，深入贯彻落实科学发展观，立足于加快推进经济社会全面发展，继续巩固农业基础地位，实施矿业富市、旅贸活市、科教兴市发展战略，初步建立创新型工业经济体系、现代化水平较高的农业体系、功能完备的现代物流体系、有效承载科学发展的生态体系、满足事业发展需求的人才体系、更加完善的人民生活保障体系，创新社会管理，努力打造对俄合作平台、现代农业基地、北疆旅游文化名城和宜居宜业幸福城市。到2016年，力争全社会固定资产投资翻两番，地区生产总值、社会消费品零售总额、全口径财政收入、城乡居民收入实现倍增；地区生产总值、人均地区生产总值、城镇居民人均可支配收入、农村居民人均纯收入、城镇人均住房建筑面积、森林覆盖率、自然保护区面积比例等指标提前实现全面小康；中低收入群体和贫困人口生活持续改善；城市化和城乡一体化水平大幅跃升，城乡环境更加优美；社会事业全面进步，民主法治持续加强，社会更加和谐美好。

市五次党代会描绘了未来五年的宏伟蓝图，政府将狠抓工作落实，打好“四张牌”，建设“一都三地”魅力黑河。

——打文化牌，打造中俄风情之都。进一步丰富独特文化内涵，明确文化定位，彰显文化个性，塑造城市特色，把黑河从对俄交流的功能城市上升为中俄风情浓郁的文化城市，在中国乃至世界叫响“中俄风情之都”品牌。

——打区位牌，构筑中俄合作高地。进一步放大区位优势，跳出黑河看黑河，站在服务全省全国的高度，构筑对俄大交通、大交流、大合作格局，在“两国一城”的创新实践中，努力建设中俄边境全方位合作示范区。

——打生态牌，建设休闲养生福地。进一步挖掘生态潜力，以市区和五大连池风景区为两极，整合旅游资源，加大对外宣传，突出火山、矿泉、界江、冰雪、境外旅游、森林湿地、绿色美食，把黑河打造成更具特色的北国休闲养生旅游目的地。

——打资源牌，培育植财生金宝地。进一步树立科学发展理念，坚持生态发展，实现绿色崛起，大力发展农林基础产业、矿产支柱产业、文化旅游特色产业、外贸物流现代服务业、新能源等战略性新兴产业“五大产业”，将资源优势尽快转化为经济优势，使黑河成为我省经济发展的亮点，成为创业者投资兴业的乐园。

各位代表，民生无小事，枝叶总关情。我们在加快经济发展的同时，将更加关注民生建设，更加重视城乡发展，更加关心困难群体，全力解决好群众就业、就医、就学、住房等方面问题，将更多的财力向民生领域倾斜，在未来五年绘就更精彩的民生画卷，让广大群众生活得更舒心、更美好。这是全市人民的热切期盼和福祉所在，更是新一届政府的神圣职责和庄严承诺！

三、2012年工作目标和主要任务

今年是新一届政府开局之年，做好今年工作至关重要。主要预期目标是，全市生产总值增长13%。全社会固定资产投资增长30%。社会消费品零售总额增长15%。全口径财政收入增长20%。对外贸易进出口总值增长15%。城镇居民人均可支配收入、农村居民人均纯收入分别增长12%和10%以上。万元地区生产总值综合能耗下降3%。着重抓好以下工作：

（一）全力推进产业项目建设，保持投资快速增长。产业项目是经济发展的原动力，必须加快项目建设，培植财源、创造财富。今年安排开复工重点项目116项，年度投资170亿元。其中，产业项目81项，年度投资84亿元，增长31%。抓开工抓投产。落实县（市、区）责任，出台考核办法，严格兑现奖惩。发挥市直部门职能，加快前期手续报批，对投资亿元以上重大项目，按季度集中会办，破解制约难题，为项目建设创造良好条件，力争早开工、早投产、早达效。市级重点项目开工率达到85%以上，投产率达到45%以上。省重点推进产业项目全部开工，力争超额完成年度计划。抓谋划抓储备。围绕“五大产业”，围绕产业链延伸，围绕重点企业吃配，谋划和生成一批科技含量高、发展潜力大、拉动能力强的大项目。强化项目储备，动态更新项目库，从差异化角度研究谋划项目，做到人无我有，人有我优。设立1000万元项目引导资金，新谋划储备重点项目达到450项。抓园区抓招商。提高园区集聚能力和投资强度，集中精力打造特色专业园区。以爱辉区、北安、孙吴、逊克和五大连池为原料基地，按照“飞地经济”模式新建市属农林产业园区，实现园区共建、产业共育、税收共享。整合五秀山俄电加工区和瑷珲对俄进出口加工基地，使之成为集进出口加工、高新技术、对俄物流为一体的新型园区。谋划筹建冶炼园区，完成选址、规划工作，以园区带动资源初加工向深度开发转型。其他县（市）也要进一步完善园区基础设施，力争有更多企业入区。按照九个“更加注重”原则，积极开展以商招商、挂职招商、园区对接招商、科研成果转化招商，努力提高招商实效，到位资金增长20%以上。出台新的招商政策，落实招商责任，动员全社会力量投身到招商引资中来，形成全员招商的浓厚氛围。

（二）大力发展现代农业，增强可持续发展能力。现代农业是农业可持续发展的必由之路，必须深化局市共建，推动现代农业大发展。必须用工业化思路谋划农业，用市场化思维经营农业，大力推进农业产业化，实现粮食增产和农民增收。农业增加值增长4%以上，粮食总产增长10%以上。调整种植结构。实施好种植业结构调整规划，大豆面积调减19%，水稻、玉米、马铃薯和经济作物实现大幅增长，扩大农作物轮作示范带规模，明年实现科学轮作，努力解决大豆重迎茬和土地药害问题。保证玉米等农作物种子供应。推进爱辉区、北安、孙吴、逊克、五大连池5个寒地水稻示范区建设，高标准抓好逊克场县共建现代农业示范基地建设。培育特色农业。大力开展森林经营，实施低质低效林综合改造，完成造林绿化37.5万亩，森林抚育50万亩以上，积极开展碳汇林业基础研究。扩大蓝靛果忍冬、北药种植规模，发展特色林产经济。提高畜牧养殖水平，力争每个县（市、区）建成一个省部级标准化示范场。加快网箱水产养殖基地建设，新增网箱3000个。设立财政扶持资金，支持企业境外粮食回运，完善农产品质量监管体系，打造中俄边境无污染农产品特色品牌。规范合作组织。发展农村专业合作社50个，力争10个纳入省“寒地黑土”千社千品工程。建设大型现代农机合作社10个。土地流转、规模经营面积分别增长10%和15%以上。加快建设农产品信息服务平台，从注重研究“田间地头”向“市场端头”转变，为农民闯市场提供信息服务。加快小城镇建设。高品位设计、高标准建设10个城乡一体化示范镇，推动公共设施和公共服务向小城镇延伸，扩大经济总量和人口规模，提高承载能力。大力实施 “三带百村”工程，建设中心村30个，加快新农村建设进程。

（三）着力壮大地方工业，提升工业经济整体素质。工业是富市强市的依托，必须加快发展特色优势产业，扩大工业经济总量。规模以上工业增加值增长16%。做大做强农林基础产业。加快爱辉区和逊克蓝莓加工、嫩江完达山鲜奶、孙吴精制糖加工、五大连池矿泉饮品、北大荒矿泉水等项目建设，扶持爱辉区中兴牧业、嫩江祥润、嵩天薯业、孙吴大果沙棘等企业发展。积极引进战略投资者，加快蓝靛果忍冬、北药产业化开发。全市龙头企业达到65户。加快发展矿产支柱产业。抓好矿产资源勘查，加快嫩江多宝山铜矿二期、上马厂和大青山岩金矿前期工作。推进嫩江铜山铜矿、逊克翠宏山铁多金属矿、东安岩金矿建设。强化采矿权和探矿权管理，搭建矿业开发平台和矿业权交易平台，促进矿业权市场健康发展。建设绿色矿山，严格监督企业履行环境保护与恢复治理义务。积极培育新能源等战略性新兴产业。大力发展水能、风能、生物质能等新能源产业，加快爱辉区金湾水电站、龙源风电三、四期工程、逊克乌二水电站、嫩江生物质发电等项目建设。积极发展新材料、新医药以及云计算等新兴产业。推进传统产业改造升级。以北方水泥为龙头，并购整合现有水泥企业，扩大生产规模。抓好黑河富宏煤矿资源整合、爱辉区振兴煤矿扩能改造，提升煤炭资源开采和综合利用水平。推进黑河粉煤灰砌块、北安洪昌节能灯改扩建项目，支持企业发展壮大。加快冶金、煤炭、建材、供热企业节能改造，全面完成节能指标。抓好中小企业成长工程，鼓励非公经济竞相发展。

（四）打造“两国一城”，推动合作共赢。“两国一城”是中俄边境地区合作的创新实践，必须坚持有胸怀、重诚信、讲共赢的原则，深化框架下各领域合作，发展外贸物流现代服务业，以大通道带动大通关，实现大开放。提高外经贸贡献率。出口抓加工，进口抓落地，依托五秀山俄电加工区和瑷珲对俄进出口加工基地，积极引进和培育进出口加工企业，增加地产品出口比重，提高外贸对地方财政贡献率。地产品出口额增长10%以上。推进重点合作项目建设。争取阿州水泥粉磨站投产，阿穆尔—黑河边境油品储运与炼化综合体项目境外工程开工，推动对俄合作取得更大成果。协调阿州解决外派劳务问题，满足合作项目用工需求。加快构建对俄大通道。建设黑河口岸物流园区，启动浮箱固冰通道连接道路、通道码头建设，推进旅检码头二期工程。加快逊克口岸建设。提高联检部门服务质量，改善通关环境，加快通关速度。敦促俄方尽快完成黑龙江大桥审批。做好北黑、绥北省管公路项目服务，推进嫩黑公路、黑嘉公路黑河至逊克段建设。打通“珠三角”、“长三角”航空通道，增加省内支线班次，构建便捷通畅的立体交通网络。深化各领域交流。密切与阿州布市交流合作，落实好政府间会谈纪要，开展经常性互访，深化民间交往，提升经贸、文化领域交流层次。办好大黑河岛冬季经贸洽谈会、第三届中俄文化大集和中俄林业生态建设学术论坛，提高黑河在全国对俄开放大格局中的影响力。

（五）促进融合互动，发展文化旅游特色产业。文化是提升旅游产业层次的核心，必须利用好丰富的文化资源，打造特色旅游景观，实现文化旅游产业共赢。接待国内外旅游者增长11%。开发文化旅游资源。充分利用好欧陆、民俗、红色、生态等旅游资源，促进文化和旅游产业有机融合。抓好中俄民族风情园续建工程，推进中俄画家村和中俄影视娱乐城建设，筹建中俄美食城和酒吧文化街区，开发黑龙江两岸风光游。加快爱辉区新生原始部落、民俗文化广场和孙吴二战遗迹旅游区建设。以市场化运作方式，策划中俄风情文艺专场演出。提升旅游产业层次。编制“中俄双子城”旅游发展规划，设立500万元文化旅游发展资金。加快五大连池风景区旅游名镇建设，推进火山博物馆、格球山景区综合开发、五大连池机场建设。黑河城市规划体验展示馆完工，大黑河岛游乐园开园。借助中俄互办“旅游年”契机，与阿州布市共同推介“中俄双子城”。加大央视等主流媒体宣传力度。扩大消费规模。积极承办高层次、大规模会议会展活动，筹建集会展、餐饮、娱乐于一体的大型文体活动场所，建设五星级酒店，提高接待服务水平，聚人气、促消费。聚集人员增长10%以上。扩大试车经济影响，增强对国内外名企吸引力。保护俄公民合法权益，严厉打击强买强卖行为，营造良好消费环境。

（六）强化规划建设管理，完善城市综合功能。城市建设是提升城市影响力的关键，必须完善服务功能，深入推进“三城联创”，全面提升各县（市）建设管理水平，让群众共享整洁舒适、优雅宁静的生活环境。健全城市规划体系。启动新一轮城市总体规划修编工作，加快近期建设规划、城乡一体化建设规划编制，完成棚户区改造控详、修详规划，完善规划体系。加强项目规划监察，严厉查处在建违规行为，维护规划权威性。推进重点工程建设。加大市政工程投入，市区新建道路、沥青罩面7条，启动南部公园建设，推进黑龙江公园四期，实施黑河机场候机厅广场和火车站前广场改造工程。加快重点区域景观亮化，完成12条街路绿化，增设大型雕塑和情景雕塑。市区污水处理厂二期、逊克污水处理厂完工，孙吴、五大连池污水处理厂二期开工，嫩江、孙吴、五大连池生活垃圾处理厂投入使用。市区生活垃圾处理厂改造完工，医疗垃圾处理厂通过省验收。争取“黑河四岛”工程立项。提升城市管理水平。建设黑河地理信息公共服务平台，开发集规划、城管、国土、林业等功能为一体的应用系统，年末试运行。王肃街、龙江路等主要路段实施重点车型“限行”，建设集中停车场3处。加强牌匾设置、渣土清运管理，整治小广告，改造架空管线。完善城市服务功能。加强社区建设，改善办公条件，实现卫生保洁进社区，城管执法进社区，开展个性化便民服务，加强老旧弃管楼物业管理。筹建老年活动场所，丰富老年人精神文化生活。推进市区沙滩浴场建设，满足群众亲水需求。建设便民候车亭24组，改善群众候车环境。

（七）加强财税金融工作，提升支持保障水平。财税金融是经济社会发展的重要保障，必须完善公共财政体系，优化金融服务环境，实现财税金融与经济社会良性互动。壮大财政实力。强化征管，确保应征税款足额入库。规范非税收入，增加可用财力。完善煤炭产销监控体系，并向非煤矿山延伸。优化支出结构，加大重点项目、科教文卫、社会保障投入。规范部门预算管理，严格控制弹性开支。积极向上争取，到位资金增长10%以上。提高金融服务水平。打造诚信黑河，改善金融生态环境。建立联席会议制度，搞好政银企对接。建立担保补偿机制，提高融资担保能力。小额担保贷款公司发展到18家，融资性担保公司发展到4家。精心培育嫩江地铁、鹿源春等企业包装上市。继续实行金融机构奖励政策，鼓励金融机构加大对重点项目和中小企业支持，为企业投放贷款额度增长15%以上。

（八）加大改善民生力度，营造和谐稳定社会环境。改善民生是政府工作的出发点和落脚点，必须时刻把群众利益放在首位，一如既往地推进民生工程建设，毫不松懈地抓好安全生产和社会稳定，让群众生活得更加幸福安宁。重视科技人才工作。大力推进科技创新，积极引进高新技术企业，转化科技成果5项。加强人才工作，引进急需紧缺人才，培养学科梯队1—3支和技能人才400人。发展社会事业。创建义务教育标准化学校22所，扎实推进校安工程，做好市职教中心晋升高职学院前期工作，积极探索与国内外高校合作办学。出台校车管理规定，配齐配足标准校车，重点时段实施校车“绿色通道”。稳妥推进公立医院改革试点，提高医疗服务质量。继续开展好“周末大舞台”、“金色田野”等活动，满足群众精神文化需求。提高“横渡黑龙江”举办层次。抓好就业和社会保障。城镇新增就业1.6万人，登记失业率控制在4.5%以内。安排小额担保贷款6000万元，培训创业人员3000人。扩大社会保障范围，失业保险实现市级统筹，全市企业养老保险覆盖人数8.3万人，新型农村社会养老保险实现全覆盖。提高保障标准，市直城镇职工基本医疗保险住院医疗费最高支付限额由4万元提高到6万元，大额医疗补助标准由2万元提高到6万元。全力维护社会稳定。高度重视安全生产，严格落实政府监管责任和企业主体责任，加强矿山、道路交通、消防、界江生产作业、危险物品及人员密集场所安全管理，坚决遏制重特大事故发生。加强森林防火工作，确保不发生重特大森林火灾。创新社会管理，推进行政复议试点，加强群众诉求平台建设，及时化解矛盾。完善社会治安防控网络，提高社会治安防控能力。加强国防后备力量建设，做好新形势下“双拥”工作。同时，进一步做好计生、老区、民族宗教、地震、侨务、妇女儿童、残疾人、人防、档案、地方史志等工作。

各位代表，我们在统筹兼顾，做好各项工作的同时，今年将全力办好 “十二件民生实事”。一是城镇居民基本医疗保险门诊费实行社会统筹，市直居民基本医疗保险住院医疗费最高支付限额由2万元提高到5.5万元，门诊大病医疗补助最高限额由5000元提高到8000元。实现低保和优抚群体医疗救助“一站式”即时结算。二是新型农村合作医疗乡镇卫生院住院最高报销比例达到85%以上，报销封顶线由5万元提高到10万元。三是将原低保低收入80周岁以上老年人高龄补贴扩大到全市所有80周岁以上老年人，建设黑河失能老人养护中心。四是改善低收入群体住房条件，市区维修老旧住宅楼300栋，解决重点优抚对象住房困难问题。五是建立健全最低生活保障标准与物价联动机制，切实减轻物价上涨对低保群体生活的影响。六是加快保障性安居工程建设，全市改造棚户区57.16万平方米，改造泥草房1.59万户，市区配建廉租住房300套。七是新建改扩建公办幼儿园16所，创建市级一类幼儿园5所。八是继续推进30周岁以下高校毕业生就业工程，就业率达到85%以上。九是设立市区蔬菜储备库，保证市场主要蔬菜品种平稳供应。十是建设市区自来水复线工程，启动城市水源地污染防治工程，为城市供水提供保障。十一是实施农村饮水安全工程，解决40个村屯饮水安全问题。十二是设置市区便民自行车租赁网点50处，便利群众出行。

四、加强政府自身建设

今年的目标任务繁重艰巨，对政府工作提出了新的更高的要求，我们肩负的责任重大，必须加强自身建设。进一步加强依法行政。自觉接受市委领导，主动向人大报告工作、向政协通报情况，加强与民主党派、工商联、无党派人士和群团组织沟通。主动接受社会公众和新闻舆论监督。严格执行重大决策公示、听证、专家咨询论证、合法性审查、社会稳定风险评估制度，提高决策科学性。认真实施行政强制法，强化行政执法监督，规范行政执法行为。进一步提高行政效能。将效率作为政府工作的生命线，已决策的事不等不靠，打足提前量，把思路和目标落实到具体行动上，体现在工作成果上。加大督办落实力度，强化跟踪问效。提升公务员素质，增强执行力和创新力。强化机关作风建设，加大行政效能投诉举报力度，严厉治庸、治懒、治散。完善电子政务网络体系。进一步改进公共服务。坚持民情恳谈制度，落实领导干部接待群众和工作联系点制度。筹建黑河行政服务中心，将与企业和群众密切相关的事项纳入中心，最大限度为企业和群众提供方便。推进办事公开，范围扩大到医院、学校、供水、供热等单位。加强应急预案宣传和演练，及时妥善处置各类突发事件。进一步推进廉洁从政。认真执行党风廉政建设责任制，强化领导干部经济责任审计和政府重大投资项目管理，坚决预防和惩治腐败。开展工程建设、产权交易、政府采购等重点领域专项治理，严肃查处各类违法违纪案件。加强政风行风建设，坚决整治损害群众利益和破坏投资环境行为。

各位代表，光荣属于过去，奋斗成就未来。让我们在市委的坚强领导下，紧紧依靠全市人民，始终保持昂扬向上的勇气、真抓实干的作风、开拓创新的激情，为全面完成各项目标任务，推动黑河经济社会跨越发展而努力奋斗！
